# Quality of Reporting of Randomised Controlled Trials of Herbal Interventions in ASEAN Plus Six Countries: A Systematic Review

**DOI:** 10.1371/journal.pone.0108681

**Published:** 2015-01-29

**Authors:** Chayanin Pratoomsoot, Rosarin Sruamsiri, Piyameth Dilokthornsakul, Nathorn Chaiyakunapruk

**Affiliations:** 1 Faculty of Public Health, Naresuan University, Phitsanulok, Thailand; 2 Center of Pharmaceutical Outcomes Research, Department of Pharmacy Practice, Faculty of Pharmaceutical Sciences, Naresuan University, Phitsanulok, Thailand; 3 School of Pharmacy, Monash University Malaysia, Jalan Lagoon Selatan, Bandar Sunway, Selangor, Malaysia; 4 School of Population Health, Public Health Building, University of Queensland, Herston, Australia; 5 School of Pharmacy, University of Wisconsin, Madison, WI, United States of America; 6 Department of Population Medicine, Drug Policy Research Group, Harvard Medical School & Harvard Pilgrim Health Care Institute, Boston, MA, United States of America; 7 Center for Pharmacoepidemiology and Pharmacoeconomic Research and Department of Pharmacy Systems, Outcomes and Policy, College of Pharmacy, University of Illinois at Chicago, Chicago, IL, United States of America; Université de Montréal, Canada

## Abstract

**Background:**

Many randomised controlled trials (RCTs) of herbal interventions have been conducted in the ASEAN Communities. Good quality reporting of RCTs is essential for assessing clinical significance. Given the importance ASEAN placed on herbal medicines, the reporting quality of RCTs of herbal interventions among the ASEAN Communities deserved a special attention.

**Objectives:**

To systematically review the quality of reporting of RCTs of herbal interventions conducted in the ASEAN Plus Six Countries.

**Methods:**

Searches were performed using PubMed, EMBASE, The Cochrane Library, and Allied and Complementary Medicine (AMED), from inception through October 2013. These were limited to studies specific to humans and RCTs. Herbal species search terms were based on those listed in the National List of Essential Medicines [NLEM (Thailand, 2011)]. Studies conducted in the ASEAN Plus Six Countries, published in English were included.

**Results:**

Seventy-one articles were identified. Thirty (42.25%) RCTs were from ASEAN Countries, whereas 41 RCTs (57.75%) were from Plus Six Group. Adherence to the recommended CONSORT checklist items for reporting of RCTs of herbal interventions among ASEAN Plus Six Countries ranged from 0% to 97.18%. Less than a quarter of the RCTs (18.31%) reported information on standardisation of the herbal products. However, the scope of our interventions of interest was limited to those developed from 20 herbal species listed in the NLEM of Thailand.

**Conclusions:**

The present study highlights the need to improve reporting quality of RCTs of herbal interventions across ASEAN Plus Six Communities.

## Introduction

Traditional Medicine is recognised as part of historical and cultural heritage in the communities of the Association of Southeast Asian Nations (ASEAN) Member States. The role and contribution of traditional medicine has been highlighted by the ASEAN Socio-cultural Community (ASCC) Blueprint under section B4: Access to healthcare and promotion of healthy lifestyles [1]. Under which, the strategic objectives were to ensure access to adequate and affordable healthcare, medical services and medicine, and promote healthy lifestyles for the people of ASEAN. Specific actions include the facilitation of research and cross-country exchange of experience in promoting the integration of safe, effective and quality Traditional Medicine, Complementary and Alternative Medicine (TM/CAM) into the national healthcare system, notably, the strengthening of the evidence base for herbal medicines and products.

ASEAN’s external relations with other nations led to the formation of an economic partnership known as the ASEAN Plus Six Group, which comprised of the members of the ASEAN plus Australia, China, India, Japan, New Zealand, and South Korea. This regional framework signifies the promotion of cooperation, prompting economic ties, increasing market scale and resource supply capacity [2].

It has been demonstrated that randomised controlled trials (RCTs) of herbal interventions seldom provide adequate methodological information, and the quality of reporting is poor [3]–[5]. Gagnier and colleagues (2006) studied the quality of reporting of RCTs of herbal medicine interventions, and observed that less than half (45%) of the consolidated standards of reporting trials’ (CONSORT) checklist items was reported across the 206 included RCTs [5]. In addition, many study reports overlooked the importance of the detailed information on the herbal products being investigated, and often, specific characteristics of the herbal interventions were omitted [6].

The significance of adequate and transparent reporting of herbal interventions cannot be overstated as it is necessary to elicit clinical significance. An Elaborated CONSORT Statement was developed to provide recommendations for the reporting of herbal medicine trials [7], which serve as an aid to editors and reviewers in assessing the internal/external validity and reproducibility of herbal medicine trials, thereby an accurate assessment of safety and efficacy can be achieved [8].

The purpose of the current study was to strengthen the evidence base for herbal medicines and products specific to the ASEAN Community. To date, no systematic assessments of the quality of reporting of RCTs of herbal interventions in ASEAN Communities have been published. Adequate reporting is considered crucial for the audience of the reports to reliably interpret the outcomes of the trials. In order to strengthen the evidence base for herbal medicines specific to the ASEAN Community as outlined in the ASCC Blueprint, specific study within the Community of ASEAN Plus six is called for. ASEAN Plus Six allowed for larger pool of the available evidence in herbal interventions as opposed to ASEAN alone. Thus, the aim of the present study was to assess the reporting quality of RCTs of herbal interventions in ASEAN Plus Six Countries using the Elaborated CONSORT Statement.

## Methods

Electronic searches for randomised controlled trials of herbal interventions from ASEAN Plus Six Countries were conducted in the following databases: PubMed, EMBASE, The Cochrane Library, and Allied and Complementary Medicine (AMED). Searches were performed from inception through October 2013, and were limited to studies specific to humans and randomised controlled trials with the use of the term “random*”.

There were seven countries within the ASEAN Plus Six Groups where herbal medicines are listed as part of the National List of Essential Medicines or National Essential Drugs List [9]–[24]. These were China [10], Japan [12], South Korea [14], Lao PDR [18], Philippines [21], Thailand [23] and Vietnam [24]. However, the lists from China and Japan were written in Chinese and Japanese, respectively. Thus, the information was not readily available in English to us at the time of the study. In addition, we were unable to access the lists from South Korea and Lao PDR. The list from Philippines included herbal medicines from the several species including *Cassia alata Linn*. Vietnam has included thyme oil and ginko biloba in the list. Thailand has a long history of traditional and herbal medicine use. Over 70 herbal medicine products are listed under the National List of Essential Medicine (2011) [23]. This list was available to us at the time of the study. Of these herbal medicine products, we were interested in the products that were developed from single herbal species instead of the multi-herb preparations as this enabled us to limit the scope of the present study. There were 20 herbal medicine products developed and manufactured from 20 herbal species that were included in the list. These 20 herbal species thus formed the basis for the search terms in the present study (**Table S1**). An electronic search strategy for a database (EMBASE) is given in **Table S2**.

### Screening

One researcher (CP) performed the searches and screened the titles and abstracts of the identified studies. The following inclusion criteria were applied:

1. The studies were of herbal interventions of interest, single or combination preparations that included any of 20 species of herbs listed in the search terms
2. The studies were randomised controlled trials in human subjects
3. The randomised controlled trials were performed in ASEAN Plus Six Countries (ASEAN: Brunei, Cambodia, Indonesia, Laos, Malaysia, Myanmar, Philippines, Singapore, Thailand and Vietnam. Plus six: Australia, China, India, Japan, New Zealand, and South Korea). Locations of the studies were assessed by the information provided under the Patients and Methods Sections of the studies. Any randomised controlled trials of herbal interventions of interest that were performed outside ASEAN Plus Six Countries were not included
4. The studies were published in English

### Data extraction and assessment of quality of reporting

Full text articles were retrieved; three reviewers (CP, RS, and PD) screened the content and performed data extraction using an electronic standardised extraction form (Data available on request). Discussions were held to resolve any disagreement or discrepancies.

For each randomised controlled trial, we extracted information on the reporting characteristics according to the 22-item Elaborated CONSORT statement of recommendations for reporting randomised controlled trials of herbal interventions [8]. An assessment of the quality of reporting was carried out whereby each CONSORT checklist item was assigned a *yes* or *no* response depending on whether the item was included in the study report under the recommended section of the report. Furthermore, additional items which were not part of the CONSORT checklist were evaluated to capture a more comprehensive picture of the study reports. These were clinical trial registration, the availability of full protocol, source of funding, authors’ affiliations, ethics approval, acknowledgement, and disclosure of conflict of interest. Data were summarised using descriptive statistics with Excel version 2010.

We also compared the differences between ASEAN and Plus Six Countries to reveal information on the number of RCTs conducted, and the overall quality of reporting between the two Groups of Countries. Chi-Square tests were performed using SPSS Software (SPSS Inc. Released 2008. SPSS Statistics for Windows, Version 17.0. Chicago: SPSS Inc.). Fisher’s Exact values were read when more than 20% of cells had expected values of less than 5.

The report of the present systematic review, where applicable, adhered to the Preferred Reporting Items for Systematic reviews and Meta-Analyses (PRISMA) statement for reporting systematic reviews and meta-analyses of studies that evaluate health care interventions [25].

## Results

**Figure 1:**
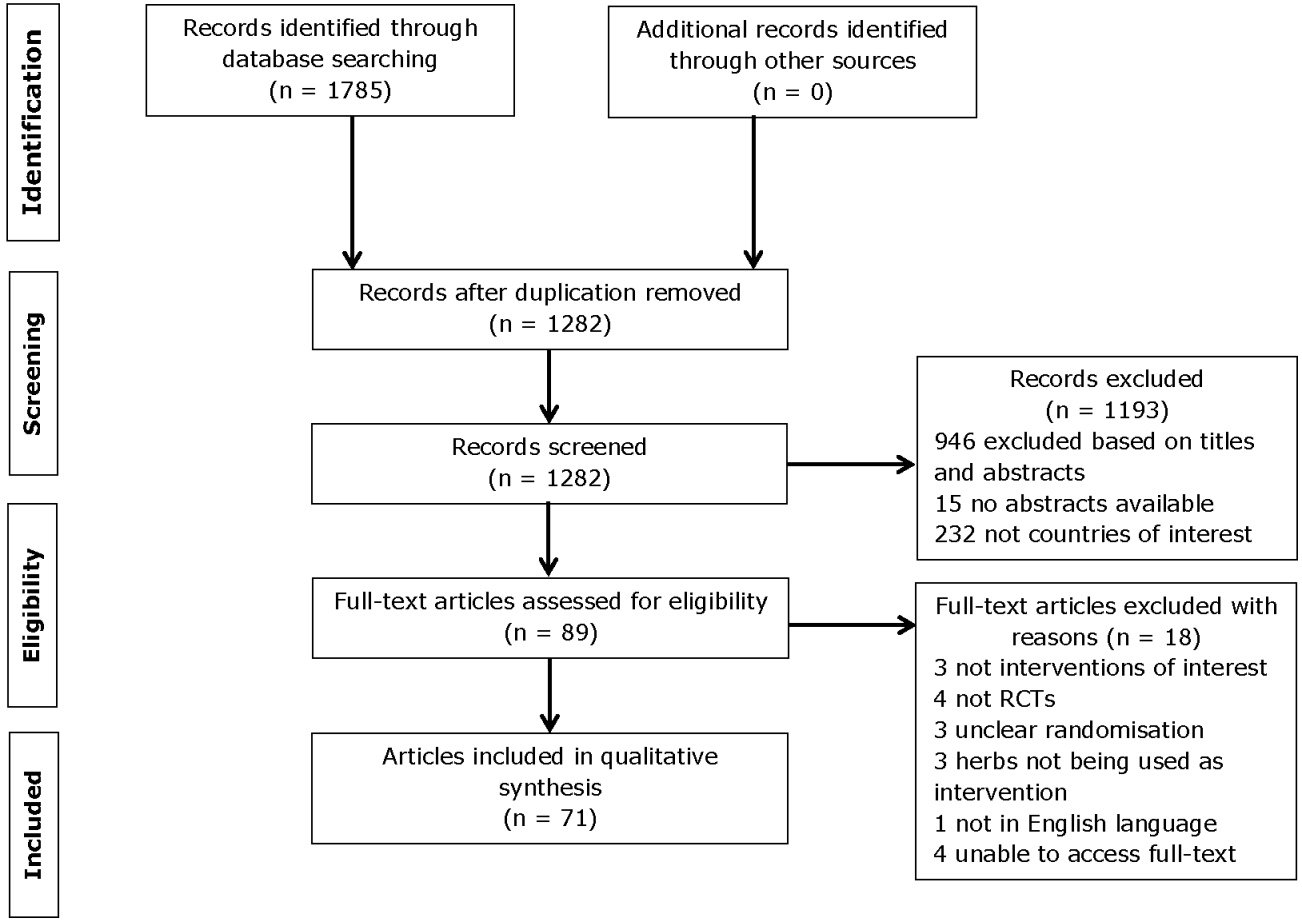
Flow chart of the identified articles, the screening and inclusion process.

The electronic searches identified a total of 1,785 records. The screening of titles and abstracts excluded 1,696 records due to duplication, studies were non-randomised controlled trials, they were not herbal interventions of interest or were of countries of interest, or they were not published in English. Eighty-nine articles were selected for full text review, subsequently a further 18 studies were excluded because they were not of herbal interventions of interest, non-RCTs, unclear randomisation, the herbs were not being used as interventions or were published in non-English language. A total of 71 articles [26]–[96] were included (Figure 1).

### General characteristics

The general characteristics of the 71 included RCT articles are presented in Table 1. The overall picture revealed that 30 (42.25%) RCTs were from ASEAN Countries, in contrast more than half (57.75%) were of the Plus Six origin. Thailand published the most RCTs of herbal interventions among the ASEAN Plus Six Countries (26 articles, 36.62%), which was followed by India (17 articles, 23.94%), and China (11 articles, 15.49%). Few studies were from Japan (5 articles, 7.04%) and South Korea (6 articles, 8.45%), while Philippines and Malaysia published the least having reported one article (1.41%) each.

**Table 1:** General characteristics of randomised controlled trials of herbal interventions in ASEAN Plus Six Countries.

Category	Description	Number (%)
		[95% CI]
**RCT by geography**	ASEAN Countries	30 (42.25)
		[0.308, 0.537]
	Plus Six Countries	41 (57.75)
		[0.463, 0.692]
**RCT by country**	Thailand	26 (36.62)
		[0.254, 0.478]
	Philippines	1 (1.41)
		[−0.013, 0.041]
	Malaysia	1 (1.41)
		[−0.013, 0.041]
	Indonesia	2 (2.82)
		[−0.01, 0.067]
	Australia	2 (2.82)
		[−0.01, 0.067]
	China	11 (15.49)
		[0.071, 0.239]
	India	17 (23.94)
		[0.14, 0.339]
	Japan	5 (7.04)
		[0.011, 0.13]
	South Korea	6 (8.45)
		[0.02, 0.149]
**Species of herb**	*Zingiber officinale*	36 (50.70)
		[0.391, 0.623]
	*Clinacanthus nutans*	1 (1.41)
		[−0.013, 0.041]
	*Curcuma longa L.*	9 (12.68)
		[0.049, 0.204]
	*Centella asiatica L.*	4 (5.63)
		[0.003, 0.11]
	*Momordica charantia L.*	2 (2.82)
		[−0.01, 0.067]
	*Senna alexandrina*	3 (4.23)
	*Cassia angustifolia*	[−0.005, 0.089]
	*Capsicum annuum L.,*	6 (8.45)
	*Capsicum frutescens L.*	[0.02, 0.149]
	*Derris scandens Benth.*	1 (1.41)
		[−0.013, 0.041]
	*Andrographis paniculata*	4 (5.63)
		[0.003, 0.11]
	*Musa paradisiaca L.*	1 (1.41)
		[−0.013, 0.041]
	*Orthosiphon grandiflorus*	1 (1.41)
		[−0.013, 0.041]
	*Garcinia mangostana L.*	3 (4.23)
		[−0.005, 0.089]
**Type of preparation**	Capsule	38 (53.52)
		[0.419, 0.651]
	Tablet	5 (7.04)
		[0.011, 0.13]
	Granule	1 (1.41)
		[−0.013, 0.041]
	Decoction	3 (4.23)
		[−0.005, 0.089]
	Cream	1 (1.41)
		[−0.013, 0.041]
	Powder	6 (8.45)
		[0.02, 0.149]
	Tea (powder)	1 (1.41)
		[−0.013, 0.041]
	Plaster	6 (8.45)
		[0.02, 0.149]
	Suspension	1 (1.41)
		[−0.013, 0.041]
	Stick	1 (1.41)
		[−0.013, 0.041]
	Semi-liquid	1 (1.41)
		[−0.013, 0.041]
	Mouthwash solution	1 (1.41)
		[−0.013, 0.041]
	Gel	1 (1.41)
		[−0.013, 0.041]
	Juice	1 (1.41)
		[−0.013, 0.041]
	Fresh part of plant	1 (1.41)
		[−0.013, 0.041]
	Essential oil	1 (1.41)
		[−0.013, 0.041]
	Standardized extract	1 (1.41)
		[−0.013, 0.041]
	Extract	1 (1.41)
		[−0.013, 0.041]
**Commercial products**	Charantia	1 (1.41)
		[−0.013, 0.041]
	Capsicum Plaster (PAS)	5 (7.04)
		[0.011, 0.13]
	Senokot	1 (1.41)
		[−0.013, 0.041]
	Johnson's Perforated CapsicumPlaster	1 (1.41)
		[−0.013, 0.041]
	Nona Roguy	1 (1.41)
		[−0.013, 0.041]
	Mangosteen Plus with EssentialMinerals	1 (1.41)
		[−0.013, 0.041]
	KalmCold	1 (1.41)
		[−0.013, 0.041]
	Herbmed	1 (1.41)
		[−0.013, 0.041]
**Commercial products**	Aller-7 (NR-A2)	1 (1.41)
		[−0.013, 0.041]
	Senna (common laxative)	1 (1.41)
		[−0.013, 0.041]
	Other commercial preparations(brand name not reported)	3 (4.23)
		[−0.005, 0.089]
	Other proprietary preparations - not marketed	3 (4.23)
		[−0.005, 0.089]
**Conditions investigated**	Laparoscopic cholecystectomy	1 (1.41)
		[−0.013, 0.041]
	Cholelithiasis	1 (1.41)
		[−0.013, 0.041]
	Non-cancer gynecologicallaparoscopy	1 (1.41)
		[−0.013, 0.041]
	Lactation post-partum	1 (1.41)
		[−0.013, 0.041]
	Maternity blues syndrome post-partum	1 (1.41)
		[−0.013, 0.041]
	Nausea and vomiting duringpregnancy	7 (9.86)
		[0.029, 0.168]
	Post-operative nausea andvomiting	6 (8.45)
		[0.02, 0.149]
	Nausea and vomiting inoncology	3 (4.23)
		[−0.005, 0.089]
	Pain after surgery	3 (4.23)
		[−0.005, 0.089]
	Osteoarthritis of knees	7 (9.86)
		[0.029, 0.168]
	Rheumatoid arthritis	1 (1.41)
		[−0.013, 0.041]
	Severe knee pain	1 (1.41)
		[−0.013, 0.041]
	Common geriatric problems(OA, T2DM, hypertension)	1 (1.41)
		[−0.013, 0.041]
	Physical performance andcognitive function in elderly	3 (4.23)
		[−0.005, 0.089]
	Senile dementia	1 (1.41)
		[−0.013, 0.041]
	T2DM	3 (4.23)
		[−0.005, 0.089]
	Dyslipidaemia	1 (1.41)
		[−0.013, 0.041]
	Wound healing in T2DM	1 (1.41)
		[−0.013, 0.041]
	Elevated ALT levels	1 (1.41)
		[−0.013, 0.041]
	Colorectal cancer	1 (1.41)
		[−0.013, 0.041]
**Conditions investigated**	Polycystic ovary syndrome	1 (1.41)
		[−0.013, 0.041]
	Uterine fibroids	1 (1.41)
		[−0.013, 0.041]
	Periphery blood flow in post-menopause	1 (1.41)
		[−0.013, 0.041]
	Lumbar disc herniation	1 (1.41)
		[−0.013, 0.041]
	Herpes zoster	1 (1.41)
		[−0.013, 0.041]
	Chronic gastritis	1 (1.41)
		[−0.013, 0.041]
	Ulcerative colitis	2 (2.82)
		[−0.01, 0.067]
	Active colitis	1 (1.41)
		[−0.013, 0.041]
	Acute diarrhoea	1 (1.41)
		[−0.013, 0.041]
	Bowel preparation	2 (2.82)
		[−0.01, 0.067]
	Urinary/renal calculi	2 (2.82)
		[−0.01, 0.067]
	Spleen/Gastric mucosa repair	1 (1.41)
		[−0.013, 0.041]
	Iron deficiency anaemia	1 (1.41)
		[−0.013, 0.041]
	Immune function	1 (1.41)
		[−0.013, 0.041]
	Upper respiratory tract infection	2 (2.82)
		[−0.01, 0.067]
	Allergic rhinitis	1 (1.41)
		[−0.013, 0.041]
	Post-operative sore throat	1 (1.41)
		[−0.013, 0.041]
	Globus hystericus	1 (1.41)
		[−0.013, 0.041]
	Cervical vertigo	1 (1.41)
		[−0.013, 0.041]
	Pruritus from mosquito bites	1 (1.41)
		[−0.013, 0.041]
	Oral/dental	2 (2.82)
		[−0.01, 0.067]
**Training/Qualification of first-author**	MD	12 (16.90)
		[0.082, 0.256]
	MD, M.Med.Sci	1 (1.41)
		[−0.013, 0.041]
	MD, PhD	4 (5.63)
		[0.003, 0.11]
	MS	1 (1.41)
		[−0.013, 0.041]
	Specialist in Anaesthesiology	1 (1.41)
		[−0.013, 0.041]
	Postgraduate Resident	1 (1.41)
		[−0.013, 0.041]
	M.S., M.Ch (Urology)	1 (1.41)
		[−0.013, 0.041]
	BScN	1 (1.41)
		[−0.013, 0.041]
	D.D.S., PhD	1 (1.41)
		[−0.013, 0.041]
	MD, MSc	1 (1.41)
		[−0.013, 0.041]
	PhD, MSc	1 (1.41)
		[−0.013, 0.041]
*Training/Qualification of first-author not reported but affiliated to*	Medical College/MedicalUniversity/Hospital	28 (39.44)
		[0.281, 0.508]
	Traditional Chinese MedicalCollege/Hospital	4 (5.63)
		[0.003, 0.11]
	University	5 (7.04)
		[0.011, 0.13]
	Research Centre/Institute	6 (8.45)
		[0.02, 0.149]
	National Institute of Ayurveda	1 (1.41)
		[−0.013, 0.041]
	Industry	1 (1.41)
		[−0.013, 0.041]
	No affiliation reported	1 (1.41)
		[−0.013, 0.041]
**Year of publication**	*Pre-2000*	
	1990	1 (1.41)
		[−0.013, 0.041]
	1991	1 (1.41)
		[−0.013, 0.041]
	1996	1 (1.41)
		[−0.013, 0.041]
	1998	1 (1.41)
		[−0.013, 0.041]
	*Post-2000*	
	2001	2 (2.82)
		[−0.01, 0.067]
	2002	1 (1.41)
		[−0.013, 0.041]
	2003	5 (7.04)
		[0.011, 0.13]
	2004	3 (4.23)
		[−0.005, 0.089]
	2005	4 (5.63)
		[0.003, 0.11]
	2006	12 (16.90)
		[0.082, 0.256]
	2007	6 (8.45)
		[0.02, 0.149]
	2008	5 (7.04)
		[0.011, 0.13]
	2009	3 (4.23)
		[−0.005, 0.089]
	2010	5 (7.04)
		[0.011, 0.13]
	2011	9 (12.68)
		[0.049, 0.204]
	2012	6 (8.45)
		[0.02, 0.149]
	2013	6 (8.45)
		[0.02, 0.149]

Total number of included randomised controlled trials: 71; 95% CI: 95% Confidence Intervals RCT: Randomised controlled trial ASEAN Countries: Brunei, Cambodia, Indonesia, Laos, Malaysia, Myanmar, Philippines, Singapore, Thailand, Vietnam; Plus Six Countries: Australia, China, India, Japan, New Zealand, South Korea OA: Osteoarthritis; T2DM: Type 2 diabetes mellitus; ALT: alanine transaminase; MD: Doctor of Medicine; M.Med.Sci: Master of Medical Sciences; PhD: Doctor of Philosophy; MS/MSc: Master of Science; M.Ch: Master of Surgery; BScN: Bachelor of Science in Nursing; D.D.S.: Doctor of Dental Surgery.

From the scope of our interventions from 20 herbal species of interest, there were 12 species of herbs that were studied within the ASEAN Plus Six Countries. *Zingiber officinale* was the most frequently investigated herb (36 articles, 50.70%), and approximately one-tenth of the reported RCTs were of *Curcuma longa L.* (9 articles, 12.68%). The least investigated herbs were *Clinacanthus nutans*, *Derris scandens Benth.*, *Musa paradisiaca L.*, and *Orthosiphon grandiflorus* (1 article, 1.41% each).

The most common types of herbal preparations were capsule (38 articles, 53.52%), powder (6 articles, 8.45%), plaster (6 articles, 8.45%), and tablet (5 articles, 7.04%). Other types of preparations were decoction, granule, cream, tea, stick, gel, and juice. Seventeen RCTs studied commercial and marketed herbal products, and three RCTs examined non-marketed proprietary preparations, whereas 51 RCTs (71.83%) investigated herbal preparations with no commercial status reported.

With regard to the training or qualifications of first-authors, a total of 18 studies (25.35%) were from authors with Doctor of Medicine (MD) qualification, of which 4 had Doctor of Philosophy (PhD). Other studies were from a specialist in anaesthesiology (1.41%), a postgraduate resident (1.41%), and a nurse with a Bachelor of Science in Nursing (1.41%), etc. However, 46 studies (64.79%) did not clearly report the training or qualifications of first-authors, but they were affiliated with Medical College/Medical University/Hospital (39.44%), Traditional Chinese Medical College/Hospital (5.63%), University (7.04%), or Research Centre/Institute (8.45%).

Most of the studies were published between 2001 and 2013 (94.37%), and only a few studies were published in the 1990s (5.63%). The highest publication records were seen in 2006 (16.90%) and 2011 (12.68%).

It was found that nausea and vomiting during pregnancy (7 articles, 9.86%), osteoarthritis of the knees (7 articles, 9.86%), and post-operative nausea and vomiting (6 articles, 8.45%) were the most studied clinical conditions. Other commonly studied conditions were type 2 diabetes mellitus, post-surgical pain, nausea and vomiting in cancer patients, and physical performance and cognitive function in the elderly (3 articles, 4.23% per each condition).

### Quality of reporting

#### Overall CONSORT checklist items

The findings of the quality of reporting are represented in Table 2. Adherence to the recommended CONSORT checklist items for reporting of randomised controlled trials of herbal interventions among ASEAN Plus Six Countries ranged from 0% to 97.18%.

**Table 2:** Quality of reporting of randomised controlled trials of herbal interventions in ASEAN Plus Six Countries.

Category	CONSORTItem	Descriptor	ASEAN	Plus Six	ASEAN PlusSix	ASEAN versus Plus Six
			N (%)	N (%)	N (%)	
			[95% CI]	[95% CI]	[95% CI]	*P*-value
**Title and Abstract**						
	**1**	How participants were allocated tointerventions	27 (38.03)	37 (52.11)	64 (90.14)	1.000
			[0.267, 0.493]	[0.405, 0.637]	[0.832, 0.971]	
		Herbal medicinal product’s Latin binomial	18 (25.35)	12 (16.90)	30 (42.25)	0.010
			[0.152, 0.355]	[0.082, 0.256]	[0.308, 0.537]	
		Part of plant used	4 (5.63)	2 (2.82)	6 (8.45)	0.233
			[0.003, 0.11]	[−0.01, 0.067]	[0.02, 0.149]	
		Type of preparation	22 (30.99)	23 (32.39)	45 (63.38)	0.136
			[0.202, 0.417]	[0.215, 0.433]	[0.522, 0.746]	
**Introduction**						
**Background**	**2**	Scientific background	28 (39.44)	39 (54.93)	67 (94.37)	1.000
			[0.281, 0.508]	[0.434, 0.665]	[0.89, 0.997]	
		Statement of reasons for the trial withreference to the specific herbal medicinalproduct being used	28 (39.44)	35 (49.30)	63 (88.73)	0.453
			[0.281, 0.508]	[0.377, 0.609]	[0.814, 0.961]	
		New indication is being investigated(if applicable)	9 (12.68)	16 (22.54)	25 (35.21)	0.432
			[0.049, 0.204]	[0.128, 0.323]	[0.241, 0.463]	
		Traditional indication is being investigated(if applicable)	19 (26.76)	17 (23.94)	36 (50.70)	0.069
			[0.165, 0.371]	[0.14, 0.339]	[0.391, 0.623]	
**Methods**						
**Participants**	**3**	Eligibility criteria for participants	29 (40.85)	37 (52.11)	66 (92.96)	0.388
			[0.294, 0.523]	[0.405, 0.637]	[0.87, 0.989]	
		Exclusion criteria	25 (35.21)	34 (47.89)	59 (83.10)	0.964
			[0.241, 0.463]	[0.363, 0.595]	[0.744, 0.918]	
		Settings and locations where data were collected	23 (32.39)	26 (36.62)	49 (69.01)	0.233
			[0.215, 0.433]	[0.254, 0.478]	[0.583, 0.798]	
**Interventions**	**4**	Precise details of the interventions intendedfor each group and how they were actuallyadministered				
	4A: Herbal medicinal product name	1.1 Latin binomial name	13 (18.31)	14 (19.72)	27 (38.03)	0.431
			[0.093, 0.273]	[0.105, 0.29]	[0.267, 0.493]	
		1.2 Common name	16 (22.54)	29 (40.85)	45 (63.38)	0.133
			[0.128, 0.323]	[0.294, 0.523]	[0.522, 0.746]	
		2.1 Proprietary product name (brand name)or the extract name	2 (2.82)	10 (14.08)	12 (16.90)	0.049
			[−0.01, 0.067]	[0.06, 0.222]	[0.082, 0.256]	
		2.2 Name of the manufacturer of the product	2 (2.82)	14 (19.72)	16 (22.54)	0.006
			[−0.01, 0.067]	[0.105, 0.29]	[0.128, 0.323]	
		3. Whether the product used is authorized(licensed, registered) in the country in whichthe study was conducted	3 (4.23)	10 (14.08)	13 (18.31)	0.121
			[−0.005, 0.089]	[0.06, 0.222]	[0.093, 0.273]	
	4B: Characteristics of the herbal product	1. The part(s) of plant used to produce theproduct or extract	18 (25.35)	20 (28.17)	38 (53.52)	0.349
			[0.152, 0.355]	[0.177, 0.386]	[0.419, 0.651]	
		2. The type of product used(e.g. raw [fresh or dry],extract)	25 (35.21)	33 (46.48)	58 (81.69)	0.759
			[0.241, 0.463]	[0.349, 0.581]	[0.727, 0.907]	
		3. The type and concentration of extractionsolvent used (where applicable)#	2 (8.33)	1 (4.17)	3 (12.50)	1.000
			[−0.027, 0.194]	[−0.038, 0.122]	[−0.007, 0.257]	
		4. The method of authentication of rawmaterial	0 (0)	2 (2.82)	2 (2.82)	0.505
			[0, 0]	[−0.01, 0.067]	[−0.01, 0.067]	
	4C: Dosage regimen and quantitative description	1. The dosage of the product and theduration of administration	26 (36.62)	38 (53.52)	64 (90.14)	0.446
			[0.254, 0.478]	[0.419, 0.651]	[0.832, 0.971]	
		2. The content of all quantified herbalproduct constituents per dosage unit form	25 (35.21)	32 (45.07)	57 (80.28)	0.580
			[0.241, 0.463]	[0.335, 0.566]	[0.71, 0.895]	
	4D: Qualitative testing	1. Product's chemical fingerprint and methods used (equipment and chemical reference standards) and who performed the chemical analysis (e.g. the name of the laboratory used)	1 (1.41)	2 (2.82)	3 (4.23)	1.000
			[−0.013, 0.041]	[−0.01, 0.067]	[−0.005, 0.089]	
		2. Description of any special testing/purity testing	2 (2.82)	2 (2.82)	4 (5.63)	1.000
			[−0.01, 0.067]	[−0.01, 0.067]	[0.003, 0.11]	
		3. Standardization	6 (8.45)	7 (9.86)	13 (18.31)	0.753
			[0.02, 0.149]	[0.029, 0.168]	[0.093, 0.273]	
	4E: Placebo/control group	The rationale for the type of control or placebo used	29 (40.85)	39 (54.93)	68 (95.77)	1.000
			[0.294, 0.523]	[0.434, 0.665]	[0.911, 1.005]	
	4F: Practitioner	A description of the practitioners (e.g., training and practice experience) who are a part of the intervention	4 (5.63)	5 (7.04)	9 (12.68)	1.000
			[0.003, 0.11]	[0.011, 0.13]	[0.049, 0.204]	
**Objectives**	5	Specific objectives and hypotheses	3 (4.23)	4 (5.63)	7 (9.86)	1.000
			[−0.005, 0.089]	[0.003, 0.11]	[0.029, 0.168]	
**Outcomes**	6	Clearly defined primary outcome measures	29 (40.85)	38 (53.52)	67 (94.37)	0.633
			[0.294, 0.523]	[0.419, 0.651]	[0.89, 0.997]	
		Clearly defined secondary outcome measures	12 (16.90)	9 (12.68)	21 (29.58)	0.100
			[0.082, 0.256]	[0.049, 0.204]	[0.19, 0.402]	
**Sample size**	7	How sample size was determined	10 (14.08)	11 (15.49)	21 (29.58)	0.553
			[0.06, 0.222]	[0.071, 0.239]	[0.19, 0.402]	
		Explanation of any interim analyses and stopping rules when applicable	3 (4.23)	0 (0)	3 (4.23)	0.071
			[−0.005, 0.089]	[0, 0]	[−0.005, 0.089]	
**Randomisation**						
**Sequence allocation**	8	Method used to generate the random allocationsequence	13 (18.31)	21 (29.58)	34 (47.89)	0.511
			[0.093, 0.273]	[0.19, 0.402]	[0.363, 0.595]	
**Allocation concealment**	9	Method used to implement random allocationsequence	9 (12.68)	12 (16.90)	21 (29.58)	0.947
			[0.049, 0.204]	[0.082, 0.256]	[0.19, 0.402]	
**Implementation**	10	Who generated the allocation sequence, whoenrolled participants, and who assignedparticipants to their groups	1 (1.41)	1 (1.41)	2 (2.82)	1.000
			[−0.013, 0.041]	[−0.013, 0.041]	[−0.01, 0.067]	
**Blinding (masking)**	11	Whether or not participants, those administering the interventions, and thoseassessing the outcomes wereblinded to group assignment (double-blind)	7 (9.86)	15 (21.13)	22 (30.99)	0.233
			[0.029, 0.168]	[0.116, 0.306]	[0.202, 0.417]	
		Single-blind	5 (7.04)	4 (5.63)	9 (12.68)	0.479
			[0.011, 0.13]	[0.003, 0.11]	[0.049, 0.204]	
**Statistical methods**	12	Statistical methods used to compare groups forprimary outcome(s)	27 (38.03)	38 (53.52)	65 (91.55)	0.692
			[0.267, 0.493]	[0.419, 0.651]	[0.851, 0.98]	
**Results**						
**Participant flow**	13	Diagram of flow of participants througheach stage	8 (11.27)	11 (15.49)	19 (26.76)	0.988
			[0.039, 0.186]	[0.071, 0.239]	[0.165, 0.371]	
		Drop-out reporting	18 (25.35)	21 (29.58)	39 (54.93)	0.463
			[0.152, 0.355]	[0.19, 0.402]	[0.434, 0.665]	
**Recruitment**	14	Dates defining the periods of recruitmentand follow-up	0 (0)	2 (2.82)	2 (2.82)	0.505
			[0, 0]	[−0.01, 0.067]	[−0.01, 0.067]	
**Baseline data**	15	Baseline demographic and clinical characteristics of each group	25 (35.21)	35 (49.30)	60 (84.51)	1.000
			[0.241, 0.463]	[0.377, 0.609]	[0.761, 0.929]	
**Numbers analysed**	16	Number of participants in each group included in each analysis	30 (42.25)	39 (54.93)	69 (97.18)	0.505
			[0.308, 0.537]	[0.434, 0.665]	[0.933, 1.01]	
		Intention-to-treat analysis	19 (26.76)	23 (32.39)	42 (59.15)	0.540
			[0.165, 0.371]	[0.215, 0.433]	[0.477, 0.706]	
**Outcomes and estimation**	17	For each primary and secondary outcome, a summary of results for each group, and the estimated effect size	30 (42.25)	41 (57.75)	71 (100)	∧
			[0.308, 0.537]	[0.463, 0.692]	[1,1]	
**Ancillary analyses**	18	Address multiplicity by reporting any other analyses performed, including subgroup analyses and adjusted analyses	2 (2.82)	2 (2.82)	4 (5.63)	1.000
			[−0.01, 0.067]	[−0.01, 0.067]	[0.003, 0.11]	
**Adverse events**	19	All important adverse events or side effects in each intervention group	20 (28.17)	27 (38.03)	47 (66.20)	0.943
			[0.177, 0.386]	[0.267, 0.493]	[0.552, 0.772]	
**Discussion**						
**Interpretation**	20	Interpretation of the results in light of the product and dosage regimen used	19 (26.76)	20 (28.17)	39 (54.93)	0.223
			[0.165, 0.371]	[0.177, 0.386]	[0.434, 0.665]	
		Sources of potential bias or imprecision	10 (14.08)	22 (30.99)	32 (45.07)	0.089
			[0.06, 0.222]	[0.202, 0.417]	[0.335, 0.566]	
**Generalizability**	21	Where possible, discuss how the herbal product and dosage regimen used relate to what is used in self-care and/or practice	20 (28.17)	30 (42.25)	50 (70.42)	0.553
			[0.177, 0.386]	[0.308, 0.537]	[0.598, 0.81]	
**Overall evidence**	22	Discussion of the trial results in relation to trials of other available products	28 (39.44)	36 (50.70)	64 (90.14)	0.691
			[0.281, 0.508]	[0.391, 0.623]	[0.832, 0.971]	

95% CI: 95% Confidence Intervals.

# There were 24 RCTs where solvent used was applicable.

∧Unable to compute (the value(s) of the variable was zero).

Fifteen CONSORT checklist items were reported, under the recommended sections, by most of the RCTs (more than 80%), including: how participants were allocated to interventions, under the abstract section (e.g., “random allocation”, “randomised”, or “randomly assigned”), statement of reasons for the trial with reference to the specific herbal medicinal product being used, the type of product used [e.g. raw (fresh or dry), extract], statistical methods used to compare groups for primary outcome(s), and discussion of the trial results in relation to trials of other available products.

Less than half of the RCTs reported the methods used to generate the random sequence allocation (item 8, 47.89%), and implement random allocation sequence (item 9 allocation concealment, 29.58%). Only one RCT completely reported the implementation of randomisation (item 10, 1.41%). In terms of blinding, approximately 31% RCTs clearly reported that they were double-blind studies with descriptions of who were blinded to group assignments (item 11). Around 27% RCTs reported diagrams of flow of participants through each stage (item 13). Albeit, 54.93% RCTs included drop-out reporting.

#### CONSORT checklist item 4

Under the CONSORT checklist item 4, where the report of precise details of the interventions intended for each group were recommended, more than half of the RCTs reported the part(s) of plant used to produce the product or extract (item 4B: characteristics of the herbal product, 53.52%), the type of product used (item 4B: characteristics of the herbal product, 81.69%), the content of all quantified herbal product constituents per dosage unit form (item 4C: dosage regimen and quantitative description, 80.28%), and the rationale for the type of control or placebo used (item 4E: placebo/control group, 95.77%).

However, only few studies presented the complete report of the method of authentication of raw material (item 4B: characteristics of the herbal product, 2.82%), the product’s chemical fingerprinting and methods used (item 4D: qualitative testing, 4.23%), and the description of any special testing/purity testing (item 4D: qualitative testing, 5.63%). Less than a quarter of the RCTs (18.31%) reported information on standardisation of the herbal products (item 4D: qualitative testing).

### ASEAN versus Plus Six Countries

Thirty RCTs (42.25%) were from ASEAN Countries, in contrast more than half (57.75%) were of the Plus Six Group. It can be clearly observed that studies from the Plus Six Group contributed larger proportions of the overall quality of reporting compared to studies from the ASEAN Countries, which could be directly related to the higher number of RCTs (41 studies versus 30 studies, respectively). This includes the method of authentication of raw material (item 4B) and product’s chemical fingerprint and methods used (item 4D).

Results of Chi-Square Tests (Table 2) showed that for most of the Elaborated CONSORT statement checklist items, there were no statistically significant differences between the quality of reporting of ASEAN and Plus Six Countries (*P* values>0.05). The checklist items that revealed statistically significant differences were 1) item 1 herbal medicinal product’s Latin binomial (*P* = 0.010); 2) item 4A name of the manufacturer of the product (*P* = 0.006); and 3) Proprietary product name (brand name) or the exact name (*P* = 0.049).

It was noted that the checklist item 1 herbal medicinal product’s Latin binomial was the only item that was reported more frequently in the RCTs of ASEAN Countries versus Plus Six Countries (P = 0.010).

### Additional items

The results of the assessment of additional items are presented in Table 3. In general, ethics approval was reported by 74.65% of the RCTs, where almost all of the RCTs from ASEAN Countries (27 of 30 studies) provided ethics approval information, in comparison with the RCTs from Plus Six Countries, only 26 of 41 studies provided such information (*P* = 0.011). Reports on clinical trial registration and disclosure of conflict of interest were not common (7.04% and 23.94%, respectively). National and University/Institute were the most common sources of funding reported (14.08% for both sources), which was followed by funding from the industry (9.86%). Most of the authors (85.92%) were affiliated to universities or national institutes. Less than half of the studies (45.07%) acknowledged contributors other than the authors. No RCTs reported any information on the availability of full clinical trial protocol.

**Table 3:** Additional items assessed for the quality of reporting of randomised controlled trials of herbal interventions in ASEAN Plus Six Countries.

Category	Description	ASEAN	Plus Six	ASEAN Plus Six	ASEAN versus Plus Six
		N (%)	N (%)	N (%)	*P*-value
**Clinical trial registration**		0 (0)	5 (7.04)	5 (7.04)	0.069
		[0, 0]	[0.011, 0.13]	[0.011, 0.13]	
**Full protocol**		0 (0)	0 (0)	0 (0)	∧
		[0, 0]	[0, 0]	[0, 0]	
**Source of funding**	National	6 (8.45)	4 (5.63)	10 (14.08)	0.304
		[0.02, 0.149]	[0.003, 0.11]	[0.06, 0.222]	
	Provincial	0 (0)	1 (1.41)	1 (1.41)	1.000
		[0, 0]	[−0.013, 0.041]	[−0.013, 0.041]	
	Ministry	1 (1.41)	2 (2.82)	3 (4.23)	1.000
		[−0.013, 0.041]	[−0.01, 0.067]	[−0.005, 0.089]	
	Foundation	0 (0)	1 (1.41)	1 (1.41)	1.000
		[0, 0]	[−0.013, 0.041]	[−0.013, 0.041]	
	Hospital	3 (4.23)	0 (0)	3 (4.23)	0.071
		[−0.005, 0.089]	[0, 0]	[−0.005, 0.089]	
	University/Institute	7 (9.86)	3 (4.23)	10 (14.08)	0.084
		[0.029, 0.168]	[−0.005, 0.089]	[0.06, 0.222]	
	Industry	4 (5.63)	3 (4.23)	7 (9.86)	0.446
		[0.003, 0.11]	[−0.005, 0.089]	[0.029, 0.168]	
	Authors	0 (0)	1 (1.41)	1 (1.41)	1.000
		[0, 0]	[−0.013, 0.041]	[−0.013, 0.041]	
**Author’s affiliations**	University/National Institute	27 (38.03)	34 (47.89)	61 (85.92)	0.502
		[0.267, 0.493]	[0.363, 0.595]	[0.778, 0.94]	
	Ministry	1 (1.41)	0 (0)	1 (1.41)	0.423
		[−0.013, 0.041]	[0, 0]	[−0.013, 0.041]	
	Municipal	0 (0)	2 (2.82)	2 (2.82)	0.505
		[0, 0]	[−0.01, 0.067]	[−0.01, 0.067]	
	Hospital	1 (1.41)	3 (4.23)	4 (5.63)	0.633
		[−0.013, 0.041]	[−0.005, 0.089]	[0.003, 0.11]	
	Industry	0 (0)	2 (2.82)	2 (2.82)	0.505
		[0, 0]	[−0.01, 0.067]	[−0.01, 0.067]	
**Ethics**	Institutional ethics committee approval	27 (38.03)	26 (36.62)	53 (74.65)	0.011
		[0.267, 0.493]	[0.254, 0.478]	[0.645, 0.848]	
	Informed consent only	1 (1.41)	3 (4.23)	4 (5.63)	0.633
		[−0.013, 0.041]	[−0.005, 0.089]	[0.003, 0.11]	
**Acknowledgement**	Contributors other than authors	18 (25.35)	14 (19.72)	32 (45.07)	0.031
		[0.152, 0.355]	[0.105, 0.29]	[0.335, 0.566]	
**Disclosure**		8 (11.27)	9 (12.68)	17 (23.94)	0.646
		[0.039, 0.186]	[0.049, 0.204]	[0.14, 0.339]	

95% CI: 95% Confidence Intervals ∧Unable to compute (the value(s) of the variable was zero).

## Discussion

Our study is the first to assess the reporting quality of RCTs of herbal interventions in ASEAN Plus Six Countries using the Elaborated CONSORT Statement. The study identified 71 RCTs of herbal interventions of interest, from the 20 herbal species listed in the NLEM (Thailand), which were conducted in the ASEAN Plus Six Countries. These RCTs were published since 1990. We generally found that none of the RCTs reported all of the CONSORT checklist items under the recommended sections. From the pool of herbal interventions of interest, more RCTs from Plus Six Countries were identified as opposed to those from ASEAN Countries. Thus explained why reports of RCTs from the Plus Six Group contributed larger proportions to the general quality of reporting. Few studies were from Australia, Japan and South Korea. This may be driven by the scope of the herbal species within the present study, which were limited to those from the list of Thailand’s NLEM.

Importantly, we highlighted that the reporting quality was poor under the checklist items 4B (characteristics of the herbal product), 4D (qualitative testing), and 4F (descriptions of practitioners). Previous study of quality of reporting of RCTs of herbal medicine interventions by Gagnier who assessed 206 reports of RCTs [5] also commented that specific characteristics of the herbal intervention were not adequately reported. However, the result of our study on diagram of flow of participants through each stage (item 13) was different from that of Gagnier [5], where we found 19% of RCTs from ASEAN Plus Six Countries reported such item versus 75.2% from Gagnier. Furthermore, all important adverse events or side effects in each intervention group (item 19) were reported in 47% of RCTs in our study compared to 64.1% of Gagnier. Another point to note is that the interpretation of the results, taking into account study hypotheses, sources of potential bias or imprecision, and the dangers associated with multiplicity of analyses and outcomes, these items were assessed as one item in Gagnier study (reported by 68.5% of RCTs). In contrast, our study assessed these items separately and the results showed that 39% reported interpretation of the results in light of the product and dosage regimen used (item 20); and 32% reported sources of potential bias or imprecision. Moreover, our study assessed the reporting of item 21 that is where possible, discuss how the herbal product and dosage regimen used related to what is used in self-care and/or practice, which was reported by 50% of RCTs from ASEAN Plus Six Countries.

In the light of ASEAN integration where a single market is aimed for 2015, harmonisation of evaluation of traditional medicines and health supplements is the main focus of interest. Special attention should be directed to ensure safety, efficacy and quality of the herbal products. Specifically, the type of extraction solvent used where applicable (in the case of the product is an extract) in addition to the plant to plant extract ratio. This is of great value as it provides the readers with information on how much of the starting plant material was required to produce a specific amount of the finished extract [8]. Furthermore, RCTs should report method of authentication of raw material, product’s constituents in terms of chemical profiling, especially special testing or purity testing as herbal medicines are often contaminated [97]. Moreover, standardisation of active chemical component(s) per dosage unit form should be reported, where applicable, to enable the audience to determine a specific effect in a clinical situation [5].

On the issue of randomisation and blinding, less than half of RCTs adequately reported information on method used to generate the random allocation sequence (47.89%), method for the implementation of random allocation sequence (29.58%), and blinding (30.99%). Other studies also found inadequate reporting of RCTs of herbal interventions [5], Traditional Chinese Medicine [98], and complementary and alternative medicine [4]. Such information is essential for readers to make judgment whether the results of the trials were subjected to bias.

Readers should be aware of the limitations within the present study. Only RCTs reported in the English language were included in the assessment. Hence, it may be considered as a language bias. In such manner, our findings can only be considered applicable to RCTs of herbal interventions reported in English. Although the selection of our databases may be considered as comprehensive, but this cannot be reciprocated for the selection or scope of our interventions of interest. Our selection was limited to those 20 herbal species listed in the NLEM of Thailand. Consequently, it may not represent the overall evidence base for ASEAN Plus Six Countries. This was clearly seen where there were more RCTs conducted in Thailand using those 20 herbal species compared to other countries within the Communities. Other countries in the ASEAN Plus Six Group may have conducted RCTs of other herbal species. Consequently, were not included in the present study as they were not part of the inclusion criteria. This may also have affected the number of the included studies, and the overall picture of the reporting quality. Nevertheless, our study represents a starting point for the assessment of RCT reporting quality of herbal interventions from ASEAN Plus Six Group.

## Conclusion

Traditional Medicine is of great importance and plays an integrative role in the ASEAN Communities. The present study highlights the need to improve reporting quality of RCTs of herbal interventions across ASEAN Plus Six Group. Efforts to support research associated with the products of herbal origins should aim to improve the quality of RCTs, including the complete and adequate reporting thereof.

## Supporting Information

Checklist S1 **PRISMA 2009 Checklist for systematic review.**
(DOC)

Table S1 **Herbal search terms used in the present study.**
(DOCX)

Table S2 **An electronic search strategy used in the EMBASE database.**
(DOCX)
