# Supplementary material for: Quality of Reporting of Randomised Controlled Trials of Herbal Interventions in ASEAN Plus Six Countries: A Systematic Review
Source: PLoS One. 2015 Jan 29;10(1):e108681. doi: 10.1371/journal.pone.0108681 (PMC4310614; doi:10.1371/journal.pone.0108681)
Supplement: Table S1 — Herbal search terms used in the present study. (DOCX) [file pone.0108681.s002.docx]

**Table S1 Herbal search terms used in the present study**

| **Number** | **Scientific name** | **Other name** |
| --- | --- | --- |
| 1 | [Musa sp.(ABB group) "Klui Nam Wa"] | Banana |
|  | [Musa sp. (ABB group ) “triploid” cv.] | Cultivated banana |
|  | *Musa sapientum L.* |  |
|  | *Musa paradisiaca L. var* |  |
| 2 | *Curcuma longa L.* | *Curcuma domestica* |
|  |  | Turmeric |
|  |  | Indian saffron |
| 3 | *Zingiber officinale* | Ginger |
|  |  | Jamaica ginger |
|  |  | Ingwer |
|  |  | *Amomum zingiber Linnaeus* |
|  |  | *Zingiber sichuanense* |
| 4 | *Senna alata L.* | *Cassia alata L.* |
|  |  | Acapulo |
|  |  | Candelabra bush |
|  |  | Candle bush |
|  |  | Ringworm bush |
| 5 | *Andrographis paniculata* | *Justicia paniculata* |
|  |  | Kariyat |
|  |  | The Creat |
|  |  | Kalmegh |
|  |  | Kal Megh |
|  |  | Kan Jang |
| 6 | *Senna alexandrina* | *Cassia acutifolia* |
|  |  | *Cassia angustifolia* |
|  |  | *Cassia obovata* |
|  |  | *Cassia senna L.* |
|  |  | Alexandria senna |
|  |  | Alexandrian senna |
|  |  | Indian senna |
|  |  | Tinnevelly senna |
| 7 | *Rhinacanthus nasutus L.* | White crane flower |
| 8 | *Piper betle L.* | Betel pepper |
|  |  | Betel vine |
|  |  | Betel leaf |
|  |  | *Chavica auriculata* |
|  |  | *C. betel L.* |
| 9 | *Centella asiatica L.* | Indian pennywort |
|  |  | Asiatic Pennywort |
|  |  | Tiger Herbal Centella |
|  |  | *Hydrocotyle asiatica L.* |
|  |  | *Trisanthus cochinchinensis L.* |
|  |  | Centella |
| 10 | *Garcinia mangostana L.* | Mangosteen |
| 11 | *Clinacanthus nutans* | *Clinacanthus burmanni* |
|  |  | *Clinacanthus siamensis Bremek* |
| 12 | *Derris scandens (Roxb.) Benth.* |  |
| 13 | *Capsicum annuum L.* | Chili Spur Pepper |
|  | *Capsicum frutescens L.* | Cayenne Pepper |
|  |  | Capsicum |
| 14 | *Zingiber montanum* | *Zingiber cassumunar* |
|  |  | *Zingiber purpureum* |
| 15 | *Hibiscus sabdariffa L.* | Jamaica Sorrel |
|  |  | Roselle |
|  |  | Rozelle |
|  |  | Red sorrel |
| 16 | *Orthosiphon aristatus* | *Orthosiphon grandiflorus* |
|  |  | Java tea |
|  |  | Kidney Tea Plant |
|  |  | Cat's Whiskers |
| 17 | *Momordica charantia L.* | Bitter Cucumber |
|  |  | Balsam Pear |
|  |  | Bitter melon |
|  |  | Bitter gourd |
|  |  | Leprosy gourd |
| 18 | *Thunbergia laurifolia* | Babbler's Bill Leaf |
|  |  | *Thunbergia grandiflora Roxb.var. laurifolia Benoist* |
| 19 | *Murdannia loriformis* | *Aneilema nudiflorum L.* |
|  |  | *bracteatum. Clarke* |
|  |  | *Aneilema bracteatum* |
|  |  | Kuntze |
|  |  | Aneilema kuntzei |
| 20 | *Vernonia cinerea L.* | Bitter bush |
|  |  | Siam weed |
