# Supplementary material for: Quality of Reporting of Randomised Controlled Trials of Herbal Interventions in ASEAN Plus Six Countries: A Systematic Review
Source: PLoS One. 2015 Jan 29;10(1):e108681. doi: 10.1371/journal.pone.0108681 (PMC4310614; doi:10.1371/journal.pone.0108681)
Supplement: Table S2 — An electronic search strategy used in the EMBASE database. (DOCX) [file pone.0108681.s003.docx]

**Table S2 An electronic search strategy used in the EMBASE database**

| **Number** | **Search strategy** |
| --- | --- |
| 1 | 'musa'/exp OR musa AND sapientum AND l. AND [humans]/lim |
| 2 | 'musa'/exp OR musa AND paradisiaca AND l. AND var AND [humans]/lim |
| 3 | 'banana'/exp OR banana AND [humans]/lim |
| 4 | cultivated AND ('banana'/exp OR banana) AND [humans]/lim |
| 5 | 'curcuma'/exp OR curcuma AND longa AND l. AND [humans]/lim |
| 6 | 'curcuma'/exp OR curcuma AND domestica AND valeton AND [humans]/lim |
| 7 | 'turmeric'/exp OR turmeric AND [humans]/lim |
| 8 | 'indian'/exp OR indian AND saffron AND [humans]/lim |
| 9 | 'zingiber'/exp OR zingiber AND officinale AND [humans]/lim |
| 10 | 'ginger'/exp OR ginger AND [humans]/lim |
| 11 | 'jamaica'/exp OR jamaica AND ('ginger'/exp OR ginger) AND [humans]/lim |
| 12 | ingwer AND [humans]/lim |
| 13 | 'amomum'/exp OR amomum AND ('zingiber'/exp OR zingiber) AND linnaeus AND [humans]/lim |
| 14 | 'zingiber'/exp OR zingiber AND sichuanense AND [humans]/lim |
| 15 | 'senna'/exp OR senna AND alata AND l. AND [humans]/lim |
| 16 | 'cassia'/exp OR cassia AND alata AND l. AND [humans]/lim |
| 17 | acapulo AND [humans]/lim |
| 18 | candelabra AND bush AND [humans]/lim |
| 19 | candle AND bush AND [humans]/lim |
| 20 | 'ringworm'/exp OR ringworm AND bush AND [humans]/lim |
| 21 | 'andrographis'/exp OR andrographis AND paniculata AND [humans]/lim |
| 22 | kariyat AND [humans]/lim |
| 23 | the AND creat AND [humans]/lim |
| 24 | justicia AND paniculata AND [humans]/lim |
| 25 | kalmegh AND [humans]/lim |
| 26 | kal AND megh AND [humans]/lim |
| 27 | kan AND jang AND [humans]/lim |
| 28 | 'senna'/exp OR senna AND alexandrina AND [humans]/lim |
| 29 | 'cassia'/exp OR cassia AND acutifolia AND [humans]/lim |
| 30 | 'cassia'/exp OR cassia AND angustifolia AND [humans]/lim |
| 31 | 'cassia'/exp OR cassia AND obovata AND [humans]/lim |
| 32 | 'cassia'/exp OR cassia AND ('senna'/exp OR senna) AND l. AND [humans]/lim |
| 33 | alexandria AND ('senna'/exp OR senna) AND [humans]/lim |
| 34 | alexandrian AND ('indian'/exp OR indian) AND ('senna'/exp OR senna) AND [humans]/lim |
| 35 | 'senna'/exp OR senna AND [humans]/lim |
| 36 | tinnevelly AND ('senna'/exp OR senna) AND [humans]/lim |
| 37 | rhinacanthus AND nasutus AND l. AND [humans]/lim |
| 38 | white AND crane AND ('flower'/exp OR flower) AND [humans]/lim |
| 39 | 'piper'/exp OR piper AND betle AND l. AND [humans]/lim |
| 40 | 'betel'/exp OR betel AND ('pepper'/exp OR pepper) AND [humans]/lim |
| 41 | 'betel'/exp OR betel AND vine AND [humans]/lim |
| 42 | 'betel'/exp OR betel AND ('leaf'/exp OR leaf) AND [humans]/lim |
| 43 | chavica AND auriculata AND [humans]/lim |
| 44 | 'centella'/exp OR centella AND asiatica AND l. AND [humans]/lim |
| 45 | 'indian'/exp OR indian AND pennywort AND [humans]/lim |
| 46 | asiatic AND pennywort AND [humans]/lim |
| 47 | 'tiger'/exp OR tiger AND herbal AND ('centella'/exp OR centella) AND [humans]/lim |
| 48 | hydrocotyle AND asiatica AND l. AND [humans]/lim |
| 49 | trisanthus AND cochinchinensis AND l. AND [humans]/lim |
| 50 | 'centella'/exp OR centella AND [humans]/lim |
| 51 | brahmi AND [humans]/lim |
| 52 | 'garcinia'/exp OR garcinia AND mangostana AND l. AND [humans]/lim |
| 53 | mangosteen AND [humans]/lim |
| 54 | clinacanthus AND nutans AND [humans]/lim |
| 55 | clinacanthus AND burmanni AND [humans]/lim |
| 56 | clinacanthus AND siamensis AND bremek AND [humans]/lim |
| 57 | c. AND nutans AND var. AND robinsonii AND benoist. AND [humans]/lim |
| 58 | 'derris'/exp OR derris AND scandens AND roxb. AND benth. AND [humans]/lim |
| 59 | 'derris'/exp OR derris AND scandens AND benth. AND [humans]/lim |
| 60 | 'capsicum'/exp OR capsicum AND annuum AND l. AND [humans]/lim |
| 61 | 'capsicum'/exp OR capsicum AND frutescens AND l. AND [humans]/lim |
| 62 | 'chili'/exp OR chili AND ('spur'/exp OR spur) AND ('pepper'/exp OR pepper) AND [humans]/lim |
| 63 | cayenne AND ('pepper'/exp OR pepper) AND [humans]/lim |
| 64 | 'capsicum'/exp OR capsicum AND [humans]/lim |
| 65 | 'zingiber'/exp OR zingiber AND montanum AND [humans]/lim |
| 66 | 'zingiber'/exp OR zingiber AND cassumunar AND [humans]/lim |
| 67 | 'zingiber'/exp OR zingiber AND purpureum AND [humans]/lim |
| 68 | 'hibiscus'/exp OR hibiscus AND sabdariffa AND l. AND [humans]/lim |
| 69 | 'jamaica'/exp OR jamaica AND sorrel AND [humans]/lim |
| 70 | 'roselle'/exp OR roselle AND [humans]/lim |
| 71 | rozelle AND [humans]/lim |
| 72 | red AND sorrel AND [humans]/lim |
| 73 | 'orthosiphon'/exp OR orthosiphon AND aristatus AND [humans]/lim |
| 74 | 'orthosiphon'/exp OR orthosiphon AND grandiflorus AND [humans]/lim |
| 75 | java AND ('tea'/exp OR tea) AND [humans]/lim |
| 76 | 'kidney'/exp OR kidney AND ('tea'/exp OR tea) AND ('plant'/exp OR plant) AND [humans]/lim |
| 77 | 'cats'/exp OR cats AND whiskers AND [humans]/lim |
| 78 | 'momordica'/exp OR momordica AND charantia AND l. AND [humans]/lim |
| 79 | 'bitter'/exp OR bitter AND ('cucumber'/exp OR cucumber) AND [humans]/lim |
| 80 | 'balsam'/exp OR balsam AND ('pear'/exp OR pear) AND [humans]/lim |
| 81 | 'bitter'/exp OR bitter AND ('melon'/exp OR melon) AND [humans]/lim |
| 82 | 'bitter'/exp OR bitter AND ('gourd'/exp OR gourd) AND [humans]/lim |
| 83 | 'leprosy'/exp OR leprosy AND ('gourd'/exp OR gourd) AND [humans]/lim |
| 84 | thunbergia AND laurifolia AND [humans]/lim |
| 85 | babblers AND bill AND ('leaf'/exp OR leaf) AND [humans]/lim |
| 86 | thunbergia AND grandiflora AND roxb. AND var. AND laurifolia AND benoist AND [humans]/lim |
| 87 | murdannia AND loriformis AND [humans]/lim |
| 88 | aneilema AND nudiflorum AND l. AND [humans]/lim |
| 89 | bracteatum AND clarke AND [humans]/lim |
| 90 | aneilema AND bracteatum AND [humans]/lim |
| 91 | kuntze AND [humans]/lim |
| 92 | aneilema AND kuntzei AND [humans]/lim |
| 93 | 'vernonia'/exp OR vernonia AND cinerea AND l. AND [humans]/lim |
| 94 | 'bitter'/exp OR bitter AND bush AND [humans]/lim |
| 95 | 'siam'/exp OR siam AND ('weed'/exp OR weed) AND [humans]/lim |
| 96 | musa'/exp OR musa AND sapientum AND l. AND [humans]/lim OR ('musa'/exp OR musa AND paradisiaca AND l. AND var AND [humans]/lim) OR ('banana'/exp OR banana AND [humans]/lim) OR (cultivated AND ('banana'/exp OR banana) AND [humans]/lim) OR ('curcuma'/exp OR curcuma AND longa AND l. AND [humans]/lim) OR ('curcuma'/exp OR curcuma AND domestica AND valeton AND [humans]/lim) OR ('turmeric'/exp OR turmeric AND [humans]/lim) OR ('indian'/exp OR indian AND saffron AND [humans]/lim) OR ('zingiber'/exp OR zingiber AND officinale AND [humans]/lim) OR ('ginger'/exp OR ginger AND [humans]/lim) OR ('jamaica'/exp OR jamaica AND ('ginger'/exp OR ginger) AND [humans]/lim) OR (ingwer AND [humans]/lim) OR ('amomum'/exp OR amomum AND ('zingiber'/exp OR zingiber) AND linnaeus AND [humans]/lim) OR ('zingiber'/exp OR zingiber AND sichuanense AND [humans]/lim) OR ('senna'/exp OR senna AND alata AND l. AND [humans]/lim) OR ('cassia'/exp OR cassia AND alata AND l. AND [humans]/lim) OR (acapulo AND [humans]/lim) OR (candelabra AND bush AND [humans]/lim) OR (candle AND bush AND [humans]/lim) OR ('ringworm'/exp OR ringworm AND bush AND [humans]/lim) OR ('andrographis'/exp OR andrographis AND paniculata AND [humans]/lim) OR (kariyat AND [humans]/lim) OR (the AND creat AND [humans]/lim) OR (justicia AND paniculata AND [humans]/lim) OR (kalmegh AND [humans]/lim) OR (kal AND megh AND [humans]/lim) OR (kan AND jang AND [humans]/lim) OR ('senna'/exp OR senna AND alexandrina AND [humans]/lim) OR ('cassia'/exp OR cassia AND acutifolia AND [humans]/lim) OR ('cassia'/exp OR cassia AND angustifolia AND [humans]/lim) OR ('cassia'/exp OR cassia AND obovata AND [humans]/lim) OR ('cassia'/exp OR cassia AND ('senna'/exp OR senna) AND l. AND [humans]/lim) OR (alexandria AND ('senna'/exp OR senna) AND [humans]/lim) OR (alexandrian AND ('indian'/exp OR indian) AND ('senna'/exp OR senna) AND [humans]/lim) OR ('senna'/exp OR senna AND [humans]/lim) OR (tinnevelly AND ('senna'/exp OR senna) AND [humans]/lim) OR (rhinacanthus AND nasutus AND l. AND [humans]/lim) OR (white AND crane AND ('flower'/exp OR flower) AND [humans]/lim) OR ('piper'/exp OR piper AND betle AND l. AND [humans]/lim) OR ('betel'/exp OR betel AND ('pepper'/exp OR pepper) AND [humans]/lim) OR ('betel'/exp OR betel AND vine AND [humans]/lim) OR ('betel'/exp OR betel AND ('leaf'/exp OR leaf) AND [humans]/lim) OR (chavica AND auriculata AND [humans]/lim) OR ('centella'/exp OR centella AND asiatica AND l. AND [humans]/lim) OR ('indian'/exp OR indian AND pennywort AND [humans]/lim) OR (asiatic AND pennywort AND [humans]/lim) OR ('tiger'/exp OR tiger AND herbal AND ('centella'/exp OR centella) AND [humans]/lim) OR (hydrocotyle AND asiatica AND l. AND [humans]/lim) OR (trisanthus AND cochinchinensis AND l. AND [humans]/lim) OR ('centella'/exp OR centella AND [humans]/lim) OR (brahmi AND [humans]/lim) OR ('garcinia'/exp OR garcinia AND mangostana AND l. AND [humans]/lim) OR (mangosteen AND [humans]/lim) OR (clinacanthus AND nutans AND [humans]/lim) OR (clinacanthus AND burmanni AND [humans]/lim) OR (clinacanthus AND siamensis AND bremek AND [humans]/lim) OR (c. AND nutans AND var. AND robinsonii AND benoist. AND [humans]/lim) OR ('derris'/exp OR derris AND scandens AND roxb. AND benth. AND [humans]/lim) OR ('derris'/exp OR derris AND scandens AND benth. AND [humans]/lim) OR ('capsicum'/exp OR capsicum AND annuum AND l. AND [humans]/lim) OR ('capsicum'/exp OR capsicum AND frutescens AND l. AND [humans]/lim) OR ('chili'/exp OR chili AND ('spur'/exp OR spur) AND ('pepper'/exp OR pepper) AND [humans]/lim) OR (cayenne AND ('pepper'/exp OR pepper) AND [humans]/lim) OR ('capsicum'/exp OR capsicum AND [humans]/lim) OR ('zingiber'/exp OR zingiber AND montanum AND [humans]/lim) OR ('zingiber'/exp OR zingiber AND cassumunar AND [humans]/lim) OR ('zingiber'/exp OR zingiber AND purpureum AND [humans]/lim) OR ('hibiscus'/exp OR hibiscus AND sabdariffa AND l. AND [humans]/lim) OR ('jamaica'/exp OR jamaica AND sorrel AND [humans]/lim) OR ('roselle'/exp OR roselle AND [humans]/lim) OR (rozelle AND [humans]/lim) OR (red AND sorrel AND [humans]/lim) OR ('orthosiphon'/exp OR orthosiphon AND aristatus AND [humans]/lim) OR ('orthosiphon'/exp OR orthosiphon AND grandiflorus AND [humans]/lim) OR (java AND ('tea'/exp OR tea) AND [humans]/lim) OR ('kidney'/exp OR kidney AND ('tea'/exp OR tea) AND ('plant'/exp OR plant) AND [humans]/lim) OR ('cats'/exp OR cats AND whiskers AND [humans]/lim) OR ('momordica'/exp OR momordica AND charantia AND l. AND [humans]/lim) OR ('bitter'/exp OR bitter AND ('cucumber'/exp OR cucumber) AND [humans]/lim) OR ('balsam'/exp OR balsam AND ('pear'/exp OR pear) AND [humans]/lim) OR ('bitter'/exp OR bitter AND ('melon'/exp OR melon) AND [humans]/lim) OR ('bitter'/exp OR bitter AND ('gourd'/exp OR gourd) AND [humans]/lim) OR ('leprosy'/exp OR leprosy AND ('gourd'/exp OR gourd) AND [humans]/lim) OR (thunbergia AND laurifolia AND [humans]/lim) OR (babblers AND bill AND ('leaf'/exp OR leaf) AND [humans]/lim) OR (thunbergia AND grandiflora AND roxb. AND var. AND laurifolia AND benoist AND [humans]/lim) OR (murdannia AND loriformis AND [humans]/lim) OR (aneilema AND nudiflorum AND l. AND [humans]/lim) OR (bracteatum AND clarke AND [humans]/lim) OR (aneilema AND bracteatum AND [humans]/lim) OR (kuntze AND [humans]/lim) OR (aneilema AND kuntzei AND [humans]/lim) OR ('vernonia'/exp OR vernonia AND cinerea AND l. AND [humans]/lim) OR ('bitter'/exp OR bitter AND bush AND [humans]/lim) OR ('siam'/exp OR siam AND ('weed'/exp OR weed) AND [humans]/lim) |
| 97 | random* |
| 98 | musa'/exp OR musa AND sapientum AND l. AND [humans]/lim OR ('musa'/exp OR musa AND paradisiaca AND l. AND var AND [humans]/lim) OR ('banana'/exp OR banana AND [humans]/lim) OR (cultivated AND ('banana'/exp OR banana) AND [humans]/lim) OR ('curcuma'/exp OR curcuma AND longa AND l. AND [humans]/lim) OR ('curcuma'/exp OR curcuma AND domestica AND valeton AND [humans]/lim) OR ('turmeric'/exp OR turmeric AND [humans]/lim) OR ('indian'/exp OR indian AND saffron AND [humans]/lim) OR ('zingiber'/exp OR zingiber AND officinale AND [humans]/lim) OR ('ginger'/exp OR ginger AND [humans]/lim) OR ('jamaica'/exp OR jamaica AND ('ginger'/exp OR ginger) AND [humans]/lim) OR (ingwer AND [humans]/lim) OR ('amomum'/exp OR amomum AND ('zingiber'/exp OR zingiber) AND linnaeus AND [humans]/lim) OR ('zingiber'/exp OR zingiber AND sichuanense AND [humans]/lim) OR ('senna'/exp OR senna AND alata AND l. AND [humans]/lim) OR ('cassia'/exp OR cassia AND alata AND l. AND [humans]/lim) OR (acapulo AND [humans]/lim) OR (candelabra AND bush AND [humans]/lim) OR (candle AND bush AND [humans]/lim) OR ('ringworm'/exp OR ringworm AND bush AND [humans]/lim) OR ('andrographis'/exp OR andrographis AND paniculata AND [humans]/lim) OR (kariyat AND [humans]/lim) OR (the AND creat AND [humans]/lim) OR (justicia AND paniculata AND [humans]/lim) OR (kalmegh AND [humans]/lim) OR (kal AND megh AND [humans]/lim) OR (kan AND jang AND [humans]/lim) OR ('senna'/exp OR senna AND alexandrina AND [humans]/lim) OR ('cassia'/exp OR cassia AND acutifolia AND [humans]/lim) OR ('cassia'/exp OR cassia AND angustifolia AND [humans]/lim) OR ('cassia'/exp OR cassia AND obovata AND [humans]/lim) OR ('cassia'/exp OR cassia AND ('senna'/exp OR senna) AND l. AND [humans]/lim) OR (alexandria AND ('senna'/exp OR senna) AND [humans]/lim) OR (alexandrian AND ('indian'/exp OR indian) AND ('senna'/exp OR senna) AND [humans]/lim) OR ('senna'/exp OR senna AND [humans]/lim) OR (tinnevelly AND ('senna'/exp OR senna) AND [humans]/lim) OR (rhinacanthus AND nasutus AND l. AND [humans]/lim) OR (white AND crane AND ('flower'/exp OR flower) AND [humans]/lim) OR ('piper'/exp OR piper AND betle AND l. AND [humans]/lim) OR ('betel'/exp OR betel AND ('pepper'/exp OR pepper) AND [humans]/lim) OR ('betel'/exp OR betel AND vine AND [humans]/lim) OR ('betel'/exp OR betel AND ('leaf'/exp OR leaf) AND [humans]/lim) OR (chavica AND auriculata AND [humans]/lim) OR ('centella'/exp OR centella AND asiatica AND l. AND [humans]/lim) OR ('indian'/exp OR indian AND pennywort AND [humans]/lim) OR (asiatic AND pennywort AND [humans]/lim) OR ('tiger'/exp OR tiger AND herbal AND ('centella'/exp OR centella) AND [humans]/lim) OR (hydrocotyle AND asiatica AND l. AND [humans]/lim) OR (trisanthus AND cochinchinensis AND l. AND [humans]/lim) OR ('centella'/exp OR centella AND [humans]/lim) OR (brahmi AND [humans]/lim) OR ('garcinia'/exp OR garcinia AND mangostana AND l. AND [humans]/lim) OR (mangosteen AND [humans]/lim) OR (clinacanthus AND nutans AND [humans]/lim) OR (clinacanthus AND burmanni AND [humans]/lim) OR (clinacanthus AND siamensis AND bremek AND [humans]/lim) OR (c. AND nutans AND var. AND robinsonii AND benoist. AND [humans]/lim) OR ('derris'/exp OR derris AND scandens AND roxb. AND benth. AND [humans]/lim) OR ('derris'/exp OR derris AND scandens AND benth. AND [humans]/lim) OR ('capsicum'/exp OR capsicum AND annuum AND l. AND [humans]/lim) OR ('capsicum'/exp OR capsicum AND frutescens AND l. AND [humans]/lim) OR ('chili'/exp OR chili AND ('spur'/exp OR spur) AND ('pepper'/exp OR pepper) AND [humans]/lim) OR (cayenne AND ('pepper'/exp OR pepper) AND [humans]/lim) OR ('capsicum'/exp OR capsicum AND [humans]/lim) OR ('zingiber'/exp OR zingiber AND montanum AND [humans]/lim) OR ('zingiber'/exp OR zingiber AND cassumunar AND [humans]/lim) OR ('zingiber'/exp OR zingiber AND purpureum AND [humans]/lim) OR ('hibiscus'/exp OR hibiscus AND sabdariffa AND l. AND [humans]/lim) OR ('jamaica'/exp OR jamaica AND sorrel AND [humans]/lim) OR ('roselle'/exp OR roselle AND [humans]/lim) OR (rozelle AND [humans]/lim) OR (red AND sorrel AND [humans]/lim) OR ('orthosiphon'/exp OR orthosiphon AND aristatus AND [humans]/lim) OR ('orthosiphon'/exp OR orthosiphon AND grandiflorus AND [humans]/lim) OR (java AND ('tea'/exp OR tea) AND [humans]/lim) OR ('kidney'/exp OR kidney AND ('tea'/exp OR tea) AND ('plant'/exp OR plant) AND [humans]/lim) OR ('cats'/exp OR cats AND whiskers AND [humans]/lim) OR ('momordica'/exp OR momordica AND charantia AND l. AND [humans]/lim) OR ('bitter'/exp OR bitter AND ('cucumber'/exp OR cucumber) AND [humans]/lim) OR ('balsam'/exp OR balsam AND ('pear'/exp OR pear) AND [humans]/lim) OR ('bitter'/exp OR bitter AND ('melon'/exp OR melon) AND [humans]/lim) OR ('bitter'/exp OR bitter AND ('gourd'/exp OR gourd) AND [humans]/lim) OR ('leprosy'/exp OR leprosy AND ('gourd'/exp OR gourd) AND [humans]/lim) OR (thunbergia AND laurifolia AND [humans]/lim) OR (babblers AND bill AND ('leaf'/exp OR leaf) AND [humans]/lim) OR (thunbergia AND grandiflora AND roxb. AND var. AND laurifolia AND benoist AND [humans]/lim) OR (murdannia AND loriformis AND [humans]/lim) OR (aneilema AND nudiflorum AND l. AND [humans]/lim) OR (bracteatum AND clarke AND [humans]/lim) OR (aneilema AND bracteatum AND [humans]/lim) OR (kuntze AND [humans]/lim) OR (aneilema AND kuntzei AND [humans]/lim) OR ('vernonia'/exp OR vernonia AND cinerea AND l. AND [humans]/lim) OR ('bitter'/exp OR bitter AND bush AND [humans]/lim) OR ('siam'/exp OR siam AND ('weed'/exp OR weed) AND [humans]/lim) AND random* AND [humans]/lim |
